# A New Measure of Decompression Sickness in the Rat

**DOI:** 10.1155/2014/123581

**Published:** 2014-05-25

**Authors:** Peter Buzzacott, Aleksandra Mazur, Qiong Wang, Kate Lambrechts, Michael Theron, Jacques Mansourati, François Guerrero

**Affiliations:** Laboratoire Optimisation des Régulations Physiologiques (ORPhy), UFR Sciences et Techniques, Université de Bretagne Occidentale, 6 avenue Le Gorgeu, CS 93837, 29200 Brest Cedex 3, France

## Abstract

In this study we assessed the reliability of a tilting-board grip score as a measure of decompression sickness in rats. In experiments using a hyperbaric compression/decompression protocol, rats were observed for signs of decompression sickness and their grip strength measured on a tilting particle board hinged to a metal frame. Angles at which rats lost grip were converted to gravitational vectors. Decreased mean grip scores following decompression were fitted to a logistic regression model with strain, age, and weight. Decrease in grip score was significantly associated with observed decompression sickness (*P* = 0.0036). The log odds ratio for decompression sickness = 1.40 (decrease in grip score). In rats with no decrease in mean grip score there was a 50% probability of decompression sickness (pDCS). This increased steadily with decreases in mean grip score. A decrease of 0.3 had a 60% pDCS, a decrease of 0.6 had a 70% pDCS, and a decrease of 2.1 had a 95% pDCS. The tilting board grip score is a reliable measure of the probability of decompression sickness.

## 1. Introduction

Decompression sickness (DCS) is protean in manifestation with symptoms ranging from mild skin rash or pain through paralysis and even death [1]. Treatment usually involves hyperbaric recompression and the rate of full recovery remains unchanged in 30 years at around 80% [2–4]. DCS is a risk for aviators, astronauts, hyperbaric doctors, nurses and patients, tunnel/caisson workers, and balloonists but the vast majority of human cases occur after diving with compressed gas. It is of greatest concern among subsistence fishermen divers in tropical regions where morbidity and mortality incidence is an order more common than in developed countries and hyperbaric treatment is often unavailable [5, 6].

Reliable diagnostic tests for DCS remain a desirable yet elusive goal. Many markers of decompression stress have been investigated, most notably postdecompression bubble grades [7–9] but also exhaled nitric oxide [10] and cutaneous and vascular blood flow [11]; however their association with the genesis of DCS remains the subject of continued investigation. Since it is both unethical and undesirable to provoke DCS in man, and as there is currently no reliable inanimate physiological alternative, animal models serve us well in this regard, most notably the humble rat.* Rattus norvegicus* is a convenient research subject, widely available, relatively inexpensive, easily handled, and with a range of physiological characteristics that are similar to those found in humans [12].

The majority of previous rat DCS research has used binary outcomes in the analysis. DCS was classified as dead or alive [13, 14] or DCS versus no DCS [15–20]. Survival time occasionally augmented the former [13, 21, 22]. Many rat studies have based the diagnosis of DCS upon observable signs such as walking difficulties [14, 18, 19, 22–29], paralysis [14, 16–19, 23–32], rolling in a rotating cage [16–19, 23–26, 28, 33], twitching/convulsions [16–19, 23–26, 28], and respiratory distress [14, 18, 23–25, 27–32]. Spinal DCS or neurological DCS has also been occasionally classified distinctly [22, 27]. In one study a weighted severity score was given according to the number of animals and perceived “severity” of the signs [34]. Objective measures have been proposed such as a walking assessment in a rotating cylindrical cage [18, 25, 33, 35, 36], bubble grades [27, 37, 38], platelet counts [27], nitric oxide [39], bronchoalveolar and/or pleural fluids [39], inflammatory mediators (thromboxane B_2_ and leukotriene E_4_) [39], interleukin-6 [30], whole blood and differential cell counts [39], neutrophils [39], EGR-1/*B*-actin ratio in the lung [20], time to lose equilibrium following ether anesthesia [40], lactic dehydrogenase (LDH), and creatine phosphokinase (CPK) plasma activity [29]. Only rarely have objective measures been correlated with subjective observer agreement. In one study observer diagnosis was compared with platelet counts [27], while another found correlation between observer diagnosis and EGR-1/*B*-actin ratio in the lung [20]. All the aforementioned measures require the death of the animal, are invasive in some way or, in the case of the rotating wheel, potentially exacerbate DCS. Bubble grades, for example, are invasive but do not require the death of the animal. For research where recovery from decompression is necessary an objective benign test for DCS in the rat is preferable.

A review of the literature suggested one of the four most common signs of DCS in the rat is difficulty walking or paralysis, particularly in the hind legs. A tilt board has been previously described for assessing recovery from induced spinal cord trauma [41]. In this study we assessed the reliability of a tilting-board grip score as a measure of decompression sickness in rats.

## 2. Methods

Rats were obtained from Janvier SAS (Le Genest St Isla, France) and housed singly or in pairs in an environmentally controlled room (temperature 21 ± 1°C, relative humidity 27% ± 16%, 12-12 h light-dark cycle). They were fed standard rat chow and water ad libitum. After at least one week of “settling in” each rat was randomly assigned to one of the experimental or control groups shown in Table 1. The range of characteristics of the rats is also presented in Table 1. These different groups were involved in various separate experiments, each with differing power and sample size requirements.

The control groups received no injections and had access to water ad libitum, as did the Wistar group. The Losartan group received 10 mg·kg^−1^·day^−1^ of Losartan in 50 mL of water for four weeks. Nifedipine was similarly administered to the Nifedipine group at 20 mg·kg-1·day^−1^. The Vitamin C group received 50 mL·day^−1^ of water containing 200 mg·L^−1^ of Vitamin C for four weeks. The Ethanol group received 50 mL·day^−1^ of water containing 1.2% Ethanol for four weeks. The Enalapril group received 10 mg·kg^−1^·day^−1^ of Enalapril in 50 mL of water for four weeks. The NaCl group received intraperitoneal injections of 5 mL water containing 0.9% NaCl at 24 hr, 12 hr, and 30 mins prior to compression. The N-acetylcysteine group was similarly administered 5 mL of water containing 100 mg·kg^−1^ of N-acetylcysteine.

**Figure 1:**
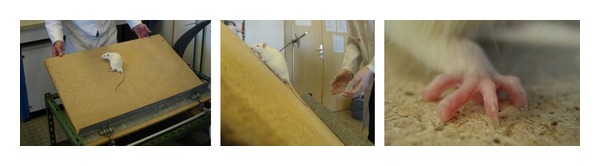
The tilting board apparatus.

A large tilting board was constructed using particle board and a hinged metal frame (Figure 1).  Table 2 presents the angles (in degrees) and gravitational vectors corresponding to the numbered scale on one side of the table. Each rat was placed on the board facing “uphill” on an angle of 10° and immediately a second researcher in front of the rat steadily lifted the front of the board at a rate of approximately 6° per second. At the first movement of the board each rat appeared to grip the surface (Figure 1), the angle steadily increased, and eventually each rat slid down the board, coming to rest at the base where a galvanised metal stop prevented the rat from leaving the apparatus. In this study no rats were previously trained or acclimatised to the test but it was apparent that the more handling each animal had endured previously, for example, when having blood pressure regularly measured, the less likely it was for the rat to urinate as a stress response. No harm befell any rat during the testing of this apparatus.

During unrelated experiments using a compression/decompression protocol shown to illicit a reliable proportion of DCS in rats [42], 165 male rats (300–500 g) were tested for grip score three times before compression/decompression. Each rat was weighed immediately prior to compression. Compression occurred in a 137-litre hyperbaric chamber (Comex, Marseille, France) using air to 1000 kPa absolute at the rate of 100 kPa per minute. Pressure (in msw) was monitored in real time using a modified dive computer (Mares, Rapallo, Italy). Maximum pressure (equivalent to 90 msw) was maintained for 45 minutes followed by decompression at 100 kPa per minute to 200 kPa. Decompression was thereafter staged with five minutes pause at 200 kPa, five minutes at 160 kPa, and 10 minutes at 130 kPa. Sixty-four rats died during or soon after decompression, leaving 101 diving rats to be observed for one hour by two researchers. Any rat displaying signs of DCS (respiratory distress or difficulty walking) were classed as having DCS. Two of these survivors were euthanized before the end of the observation period to relieve pain. In every other case of DCS the affected rat would soon recover and by the end of the observation period would be indistinguishable from the rats classed as not having DCS. This has been previously described [43].

60 minutes following decompression each rat was again scored on the tilting board three times. The remainder of this paper considers these surviving rats only (*n* = 101) and excludes the deceased. All experiments were approved by the French Ministry of Agriculture and the Universite de Bretagne Occidentale animal research ethic committee and complied with the Guide for the Care and Use of Laboratory Animals published by the US National Institutes of Health [44].

## 3. Analysis

Characteristics of each rat were entered into a Microsoft EXCEL spreadsheet including weight, strain, age, three predive grip scores, three postdive grip scores, and postdecompression DCS status. Data were imported into SAS ver 9.3 (SAS, Cary, North Carolina). Differences between the mean pre- and postdive scores were calculated (nb. increases in score were considered equal to a decrease of 0). A binary, conditional logistic regression model (1) was constructed for surviving rats (probability of DCS vs: noDCS). Backwards elimination removed nonsignificant variables. Significance was accepted at *P* ≤ 0.05. Potential interactions between independent variables and weight were also tested for significance. Consider

(1) DCSij=β0+β1Weightij+β2Straini+β3Ageij +β4Dmeanij,

where DCS_*ij*_ is the logit of the odds ratio of DCS, Ln[*p*/(1 − *p*)], and *p* is the probability of DCS in any particular rat *i*, on any particular day *j*. Values for DCS status were 0 = asymptomatic, 1 = alive for one hour but with an observed degree of temporary paralysis or respiratory distress. Weight = the weight in grams, Strain is the strain of the rat, Age is the age in weeks, and Dmean is the decrease in mean measure of grip strength between before and after the experiment (in m·s^−2^). Group was included as the conditional (experimental group) stratification variable.

## 4. Results

The distribution of DCS, weight, and tilt-test scores between control groups, treatment groups, and overall is presented in Table 3.

**Figure 2:**
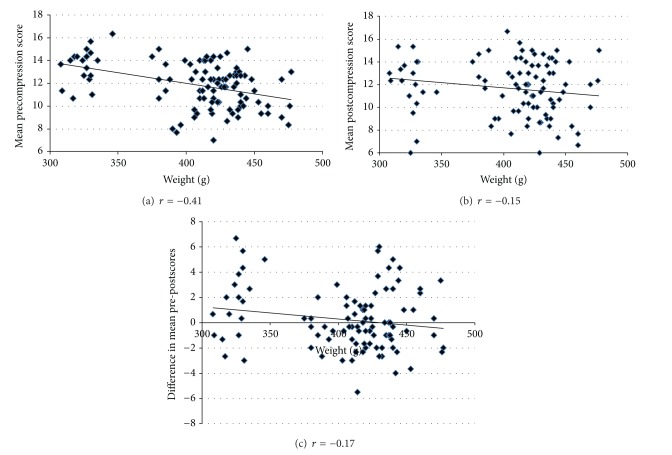
Correlation between weight and mean predive, postdive, and differential scores (*n* = 101).

As can be seen in Figures 2(a), 2(b), and 2(c) correlation with weight was −0.41 for mean predive score, −0.15 with mean postdive score, and −0.17 with postdive difference in mean scores.

In this study neither strain (*P* = 1.0), age (*P* = 0.99) nor weight (*P* = 0.90) was associated with a decrease in mean tilt-board score between pre- and postcompression. After elimination of these variables the decrease in mean tilt-board score between pre- and postcompression was solely significantly associated with DCS status (*P* = 0.0036). The relationship between decrease in mean score and probability of DCS is shown in (2). Probabilities of DCS associated with decreases in grip strength are shown in Table 4. Consider

(2) $$\begin{array}{c} \text{Ln}\left[\frac{p\text{DCS}}{\left(1-p\text{DCS}\right)}\right]=1.40\;\left(\text{decrease}\;\text{in}\;\text{mean}\right),\\ \text{if}\;\text{Ln}\left[\frac{p}{\left(1-p\right)}\right]=B,\\ \text{then}\;p=\frac{e^{B}}{\left(1+e^{B}\right)}=\frac{1}{\left(1+e^{-B}\right)}. \end{array}$$

Though the vector of downward acceleration (gravity) was used in the regression model, (it is the linear transform of the raw scores and was, therefore, the most appropriate), there was a Pearson correlation coefficient between the before and after difference in mean raw scores and mean difference in transformed vectors of 0.99. Mean scores and before to after decreases are given by DCS status in Table 5.

## 5. Discussion

The stand-out advantages of this diagnostic tool are that it is benign and that it appears to work irrespective of weight, at least in the range tested in this study (300–500 g). Any rat showing a decrease in mean grip score is at increased probability of DCS. This test is particularly useful for ranking otherwise asymptomatic rats after a period of observation during which almost all visual signs of DCS resolve. One of the three guiding principles in the Guide for the Care and Use of Laboratory Animals is that of Refinement, where experimental procedures are increasingly refined to improve the wellbeing of all animals involved [44]. This diagnostic test is the first reported with the aim of benignly diagnosing DCS in asymptomatic rats. Compression/decompression protocols exist whereby DCS is evoked in rats without a single rat dying [18]. If such “mild” DCS could be reliably diagnosed among visually asymptomatic rats then research into DCS may not necessarily utilise dead versus alive binary models of DCS. A reliable diagnostic scale should also lead to a reduction in the number of rats required to detect differences between groups in certain experiments.

It is likely that the tilting board grip-score described in this paper may be more or less reliable if used with different dive protocols. Our compression/decompression protocol has been shown to target slower, fattier tissues with half-times greater than 27 minutes. Females have not yet been tested on the tilting board, nor rats older than 13 weeks.

When using this model to estimate the probability of DCS an allowance should be made for the effect of any treatment upon the tilting-board scores of the experimental rats. In this study the effect of the various treatments varied considerably both upon the probability of DCS and its relationship with decreasing tilting board score. Nevertheless, among apparently asymptomatic rats the tilting board grip score offers a means to rank decompressed rats by an estimate of the probability they have DCS. We propose the utility of this diagnostic test will likely be optimised when used in conjunction with other complimentary, systemic markers of DCS. Further objective, benign diagnostic tests are under development.

## Figures and Tables

**Table 1:** Characteristics of each experimental group tested on the tilting board.

Experimental group		Strain^a^	Age (wks) $\overset{-}{y}$ (SD)	Weight (g) $\overset{-}{y}$ (SD)
Control groups	(*n* = 55)	S-D	11.3 (0.6)	439 (32)
Wistar, no treatment	(*n* = 20)	W	11.0 (0.0)	325 (12)
Losartan	(*n* = 15)	S-D	12.0 (0.0)	433 (13)
Nifedipine	(*n* = 15)	S-D	12.0 (0.0)	432 (19)
Vitamin C	(*n* = 15)	S-D	12.0 (0.0)	422 (13)
Ethanol	(*n* = 15)	S-D	12.0 (0.0)	433 (15)
Enalapril	(*n* = 15)	S-D	12.0 (0.0)	434 (15)
NaCl 0.9%	(*n* = 7)	S-D	11.0 (0.0)	402 (19)
N-acetylcysteine	(*n* = 8)	S-D	11.0 (0.0)	399 (18)
Overall	(*n* = 165)	S-D + W	11.5 (0.5)	417 (42)

^a^S-D: Sprague-Dawley and W: Wistar.

$\overset{-}{y}$: Sample mean.

SD: Standard deviation.

**Table 2:** Angles and gravitational vectors associated with each tilt table score.

Score	0	1	2	3	4	5	6	7	8	9	10	11	12	13	14	15	16	17	18	19	20
Angle (°)	10	13	15	18	20	21	23	25	27	30	34	36	38	40	42	44	47	50	52	55	57
Vector (m·s^−2^)^a^	1.7	2.2	2.5	3.0	3.4	3.5	3.8	4.1	4.4	4.9	5.5	5.8	6.0	6.3	6.6	6.8	7.2	7.5	7.7	8.0	8.2

^a^Vector = sin⁡⁡*θ*° × 9.8   m·s^−2^.

**Table 3:** Decompression status, weight, and grip scores by experimental status and overall.

DCS status		Control groups^a^ (*n* = 45)	Treatment groups (*n* = 56)	Overall (*n* = 101)
NoDCS	*n* (%)	29 (64)	46 (82)	75 (74)
MildDCS	*n* (%)	16 (36)	10 (18)	26 (26)
Weight (grams)	$\overset{-}{y}$, (SD)	390 (57)	421 (20)	407 (44)
Mean prescore	$\overset{-}{y}$, (SD)	12.4 (2.0)	11.5 (1.9)	11.9 (2.0)
Mean postscore	$\overset{-}{y}$, (SD)	11.4 (2.7)	11.9 (2.6)	11.6 (2.7)
Decrease pre-post^b^	$\overset{-}{y}$, (SD)	1.6 (2.0)	0.7 (1.3)	1.1 (1.7)

^a^Includes Wistar, which had no treatment.

^b^Increased grip scores were not included; therefore, the decrease is greater than the mean difference.

$\overset{-}{y}$: sample mean.

SD: standard deviation.

**Table 4:** Probability of DCS associated with decrease in mean grip strength.

Decrease in mean grip score (m·s^−2^)	0	0.3	0.6	0.9	1.2	1.5	1.8	2.1	2.4
Probability of DCS	0.50	0.60	0.70	0.78	0.84	0.89	0.93	0.95	0.97

**Table 5:** Weight and tilt-test scores by decompression sickness status and overall.

Group		DCS = 0 (*n* = 75)	DCS = 1 (*n* = 26)	Overall (*n* = 101)
Weight (grams)	$\overset{-}{y}$, (SD)	406 (41)	411 (51)	407 (44)
Mean prescore	$\overset{-}{y}$, (SD)	11.9 (2.0)	11.9 (2.1)	11.9 (2.0)
Mean postscore	$\overset{-}{y}$, (SD)	12.1 (2.5)	10.4 (2.8)	11.6 (2.7)
Decrease pre-post^a^	$\overset{-}{y}$, (SD)	0.7 (1.3)	2.1 (2.2)	1.1 (1.7)

$\overset{-}{y}$: sample mean.

SD: standard deviation.

^a^Increased grip scores were not included; therefore, the decrease is greater than the mean difference.
